# Saturated fat replacement in short dough biscuits with HPMC and lecithin stabilised nanoemulsions

**DOI:** 10.1038/s41538-023-00202-5

**Published:** 2023-06-07

**Authors:** Jansuda Kampa, Stephanie P. Bull, Antonio Signorello, Richard A. Frazier, Julia Rodriguez-Garcia

**Affiliations:** Department of Food and Nutritional Sciences, University of Reading, Whiteknights, Reading RG6 6DZ UK

**Keywords:** Structural properties, Polymers

## Abstract

Biscuits contain high proportions of saturated fats, which could lead to an adverse health effect. The objective of this study was to study the functionality of a complex nanoemulsion (CNE), stabilised with hydroxypropyl methylcellulose and lecithin, when used as a saturated fat replacer in short dough biscuits. Four biscuit formulations were studied including a control (butter) and three formulations where 33% of the butter was replaced with either extra virgin olive oil (EVOO), with CNE, or with the individual ingredients of the nanoemulsion added separately (INE). The biscuits were evaluated by texture analysis, microstructural characterisation, and quantitative descriptive analysis by a trained sensory panel. The results showed that incorporation of CNE and INE yielded doughs and biscuits with significantly higher (*p* < 0.05) hardness and fracture strength values than the control. The doughs made of CNE and INE showed significantly less oil migration during the storage than EVOO formulations, which was confirmed by the confocal images. The trained panel did not find significant differences in crumb density and hardness on the first bite among CNE, INE and the control. In conclusion, nanoemulsions stabilised with hydroxypropyl methylcellulose (HPMC) and lecithin can work as saturated fat replacers in short dough biscuits, providing satisfactory physical characteristics and sensory attributes.

## Introduction

Biscuits are very popular bakery products among consumers due to their convenient on-the-go format and palatability^1,2^. Biscuits can be defined as a cereal based product with a moisture content of less than 5 %^3^; short dough biscuits specifically contain higher fat and sugar content than other biscuits such as hard developed biscuits^4^. The main functionalities of fats in baked products are to impart shortening, richness, and to improve flavour and mouthfeel^5^. The typical fat products used in bakery goods include butter, margarine, shortenings, and palm oil^3,4,6^, which lead to a high content of saturated fatty acids and trans fatty acids in the products. A high consumption of saturated fat increases risk of cardiovascular diseases (CVD) and coronary heart disease (CHD) events, impacting overall health and quality of life^7–9^. This set of evidence corroborates the link between diet and health and has urged governments to set strategies to reduce the average contribution of saturated fatty acids to total dietary energy; in UK a policy restricting the promotion of foods high in fat, sugar and salt was recently issued, aiming to favour healthier options^10^.

Fat replacers are mainly categorised into three groups including lipid, protein, and carbohydrate based-fat replacers according to their chemical composition^11,12^. Carbohydrate-based fat replacers are the largest group of fat replacers^13^; recently, guar gum, polydextrose, inulin, and hydroxypropyl methylcellulose (HPMC) have been used in biscuits^14,15^. However, the evidence indicates that substitution of saturated fats with carbohydrates was associated with increase in CHD events; while substitution of saturated fats with polyunsaturated fatty acids (PUFA) and/or monounsaturated fatty acids (MUFA) has a beneficial impact on CVD and CHD events, levels of serum LDL cholesterol and markers of glycaemic control^7^.

In recent years, there have been a number of studies focused on using PUFA- and MUFA-rich vegetable oils, such as sunflower oil, olive oil, canola oil, and rapeseed oil, as saturated fat replacers in bakery products such as biscuits^16–21^. The reformulation strategies studied included replacement by bulk using sunflower oil^20,21^, adding the oil in structured oleogels^17–19^, and mixing the oils with solid fats, such as cocoa butter and shea butter, and fibres to form structured emulsions^16^. Although these studies showed a reduction of saturated fat, there was a lower reduction of the total amount of fat, and cookies containing these saturated fat replacers had higher spread value and harder texture than the control^20,21^. Cookies prepared with carnauba wax or candelilla wax oleogels had slightly harder texture and higher spread than samples prepared with shortening^18^. Other studies have investigated the incorporation of polysaccharides into oil-in-water emulsions as fat replacers in biscuits^22–25^ to reduce saturated fat and total fat content^23^. A study^25^ reported that an emulsion filled gel based on inulin and extra virgin olive oil could replace 50% of butter in shortbread cookies, resulting in a good texture and high consumer acceptance. However, the fat-reduced biscuits presented higher spreadability and required more force to break than control biscuits^23,24^. Furthermore, the increasing level of unsaturated fatty acids in a fat replacer can lead to greater lipid oxidation due to autoxidation of the oil^26^ causing problems of shelf-life and rancidity in the end product^27^.

Nanoemulsions with oil droplet diameter of less than 200 nm^28^ could provide several potential benefits in food processing towards a strategy to reduce saturated fat and total fat in food products. These include improving physical stability to gravitational separation of the fat phase in the matrix^29^, changing the physical properties and sensory perception of the product^30,31^, and improving physicochemical stability of functional compounds or of highly unsaturated oils^28,32^. Moreover, the formation, stability and functional properties of emulsions can be improved using combinations of emulsifiers^33^, which may have different characteristics such as surface activity and thickening ability. Soy lecithin has been widely used as emulsifying agent in the food industry^34–36^. It has been reported^22^ that biscuits made with high-oleic sunflower oil and lecithin possessed a breaking strength similar to the control biscuit made with shortening, and significantly improved sensory quality. HPMC is a carbohydrate polymer with surface activity, high swellability, and thermal gelation properties^37,38^. When the polymer is primarily in the solution phase it has been shown to develop a three-dimensional network that compartmentalises the continuous aqueous phase and immobilises the oil globules, resulting in a dense matrix of stable fat globules^23^. In a previous study, the authors have shown that the combination of lecithin and HMPC had a positive effect on the rheological properties of nanoemulsions, as these showed similar firmness and spreadability values to butter, suggesting that they will respond with a similar behaviour when used in mixing and dough sheeting operations^39^. In addition, these nanoemulsions have shown better physical and lipid oxidative stability of the emulsions during storage, due to (i) the formation of a thicker protective layer of lecithin and HPMC adsorbed in the interface of the oil droplets, and (ii) the formation of a stronger network in the continuous phase^39,40^. This structural functionality could help to improve the stability of the fat phase in biscuits formulated with saturated fat replacements made of oils high in PUFA and MUFA. Although nanoemulsions have been studied in food science for more than one decade, in this study a strategy is proposed through the incorporation of two emulsifiers, such as lecithin and HPMC,,to improve nanoemulsion functionality as a saturated fat replacer in short dough biscuits, specifically to improve the product nutritional fatty acids profile, texture and sensory properties. The objective of this study was to investigate the potential of a saturated fat replacer formulated as nanoemulsion made of extra virgin olive oil in water stabilised with lecithin and HPMC on the physical characteristics, microstructure and sensory attributes of short dough biscuits.

## Results and discussion

### Dough characteristics

#### Texture: sphere penetration measurement

Results from the sphere penetration test are shown in Table 1 and demonstrate that all doughs were significantly different (*p* < 0.05) to one another. Some of the main factors to consider when evaluating the functionality of a fat system in a biscuit dough are the colloidal composition, ratio of the solid to the liquid fat phase and the crystal structure of the solid fats^5,21,41^. Differences in the composition and the structure of butter and nanoemulsions will govern their rheological and textural properties, and thus their behaviour during manufacturing. Butter is a water-in-oil emulsion consisting of crystallised fat in a continuous phase in which water droplets are dispersed; in this system, the fat crystal network will define its rheological properties^41^. The nanoemulsion are an oil-in-water emulsion with lecithin stabilising the dispersed phase (oil) and HPMC providing a gel-like structure to the continuous aqueous phase; thus, the chemical composition and mechanical properties of the HMPC will govern the rheological properties of this system. the effect of different fats in the overall dough texture is determined by the consistency of the fat and its solid fat content^21^. Among the fat systems used in this study, EVOO had the lowest solid fat content thus producing the softest (*p* < 0.05) dough. It has been observed previously that the use of highly unsaturated fats in biscuits resulted in doughs with softer texture^18,42^.

**Table 1:** Hardness and oil migration of biscuit doughs (D-).

Formulations	Hardness (N)	Oil migration (%)	
		18 °C	30 °C
D-Control	2.42^c^ ± 0.1	5.35^b^ ± 0.66	9.11^c^ ± 0.73
D-EVOO	0.74^d^ ± 0.05	12.69^a^ ± 1.18	19.19^a^ ± 1.53
D-CNE	2.88^b^ ± 0.15	6.51^b^ ± 1.58	10.99^b^ ± 1.45
D-INE	3.44^a^ ± 0.52	5.88^b^ ± 2.54	11.43^b^ ± 2.17

Values are reported as means ± standard deviations. Means in the same column without a common superscript letter (^a-d^) are significantly different (*p* < 0.05).

D-Control is the dough made with butter; in D-EVOO, 33% of the butter was replaced with Extra Virgin Olive Oil; D-CNE has 30% less saturated fat by replacing butter with a Complex Nano-Emulsion; D-INE has 30% less saturated fat by replacing butter with the individual ingredients of the complex nanoemulsion.

The replacement of butter with the CNE led to dough pieces (D-CNE) with significantly higher (*p* < 0.05) hardness values than the D-control. Although in a previous study, the authors reported similar firmness values for CNE and butter at similar concentrations^39^. The reduction of the fat content in the system may have hindered some of the main functionalities of fat in a dough system, principally lubrication and aeration^5^. During mixing fat surrounds sugar and flour particles, breaking the continuity of the protein-starch matrix and reducing gluten development^5,43^. When less fat is present in the system, water has easier access to flour proteins, hydrating them and creating a cohesive and extensive matrix^5^, thus yielding harder doughs. When the ingredients of the emulsion were added individually during the mixing process the hardest (*p* < 0.05) dough was obtained (D-INE). When adding the HMPC as a dry ingredient, its formation of an entangled polymer solution within the dough matrix may have been inhibited due to limited time and shearing forces during mixing. The water added for the formation of the HMPC solution was freely available to interact with other ingredients, such as flour particles, thus higher dough hydration over dough shortening effect could have taken place in D-INE.

#### Oil migration

When the fat consistency is very soft or liquid at typical dough manufacturing temperatures, it can cause problems of oiling out^44^. Oil migration indicates weak interactions between the fat phase and all the other ingredients^19^. The migration of the fat phase outside the surface of the dough was examined and results are shown in Table 1. D- Control, D-CNE and D-INE showed no significant differences (*p* > 0.05) in oil migration at 18 °C. However, D-EVOO showed the highest oil migration from its surface (*p* < 0.05); more than double the quantity of oil migrated from D-Control. These results were expected due to the use of liquid fat to replace of butter without using any other structuring strategy that could give more stability to the liquid fat in the dough. In fact, the addition of HPMC and lecithin in the dough formulation, as a pre-prepared emulsion or as individual ingredients, stabilised successfully the oil in the biscuit doughs. When adding the CNE, the oil was emulsified by the combination of lecithin and HMPC in nanodrops in a continuous aqueous phase of entangled HMPC, which was confirmed by the confocal images as shown in Fig. 2. Confocal micrographs of biscuit doughs emulsions made of sunflower oil and HMPC as shortening replacer showed that the oil was dispersed homogeneously in a continuous phase of proteins, carbohydrates and dispersed starch granules^23^. However, the efficacy of the emulsifier mix in the stabilisation of the oil in the dough matrix was significantly (*p* < 0.05) decreased at higher storage temperature (30 °C). This could be because there was a decrease in the viscoelasticity of the HMPC solutions or emulsions due to a thermal softening effect involving inter and intra-molecular hydrogen bonds between HPMC molecules^45^. It has also been reported that at temperatures from 30 °C to 90 °C the hydrophobic headgroup of lecithin molecules changed its shape, thereby inducing oil droplet coalescence^46^. These destabilisation mechanisms, together with the increase in volume and movement of the fat phase at higher temperatures could have led to oil droplet aggregation and diffusion to the surface of the biscuit dough.

### Biscuit characteristics

#### Weight loss during baking (WL), moisture and water activity (*a*_w_)

Weight loss, moisture and water activity values of the biscuits are presented in Table 2. B-EVOO lost significantly more weight during baking (*p* < 0.05) resulting in a final biscuit with lower moisture and a_w_ values. During baking, water is available to solubilise sugars and gives place to a rich, fatty, sugary liquid viscous enough to contain the water vapour at initial stages of baking^3^. Then, the viscosity of the continuous network increases and the protein film formed is set, defining the width of the biscuit; then gases are released (carbon dioxide and water vapour) and towards the end of the baking the height of the structure collapses^3,5^. The effects of replacing butter by EVOO in WL and moisture could be explained by the distribution of the oil in the dough and its effects on water distribution and availability during baking. During mixing, oil gets dispersed as fine droplets that are significantly less effective in imparting the shortening functionality than plastic fats do^21^. When fat is poorly distributed in the dough, flour particles are more readily available to be hydrated^21^. It is then hypothesised that, when water is interacting with flour particles in the dough, instead of forming the syrupy phase the rate of evaporation during baking is higher, as it has been shown for B-EVOO.

**Table 2:** Weight loss during baking (WL), moisture, water activity (*a*_w_), spreadability index (SI), hardness and fracturability of the biscuits (B-).

Biscuits	WL (%)	Moisture (%)	*a*_w_	SI (mm)	Fracture strength (N)	Fracturability (mm)
B-Control	13.07^b^ ± 0.58	4.14^ab^ ± 0.38	0.49^a^ ± 0.04	3.32^b^ ± 0.04	27.31^c^ ± 8.83	38.00^a^ ± 0.21
B-EVOO	15.33^a^ ± 0.77	3.48^b^ ± 0.53	0.36^b^ ± 0.03	3.73^a^ ± 0.16	18.37^d^ ± 5.53	37.24^b^ ± 0.32
B-CNE	13.20^b^ ± 1.19	4.78^a^ ± 1.01	0.51^a^ ± 0.08	3.31^b^ ± 0.14	43.33^b^ ± 10.38	38.32^a^ ± 0.31
B-INE	13.23^b^ ± 0.97	4.99^a^ ± 0.89	0.50^a^ ± 0.06	3.21^b^ ± 0.14	50.86^a^ ± 16.47	36.57^c^ ± 1.07

Values are reported as means ± standard deviations. Values in parentheses are the standard deviation. Means in the same column without a common superscript letter (^a-d^) are significantly different (*p* < 0.05).

B-Control is the biscuit made with butter; in B-EVOO, 33% of the butter was replaced with Extra Virgin Olive Oil; B-CNE has 30% less saturated fat by replacing butter with a Complex Nano-Emulsion; B-INE has 30% less saturated fat by replacing butter with the individual ingredients of the complex nanoemulsion.

The incorporation of emulsifiers, such as lecithin and HMPC helped in the retention of water during baking, giving final biscuits with similar WL, moisture and *a*_w_ than B-Control. When HPMC is dispersed in water, hydrogen bonds form between the hydroxyl groups in HPMC and water^47,48^. Then during baking, there is an increase of strong hydrophobic interactions between HPMC chains that gave place to a sol-gel transition^49,50^, which will have retained more water within the biscuit systems (B-CNE and B-INE).

#### Spreadability Index (SI)

**Fig. 1:**
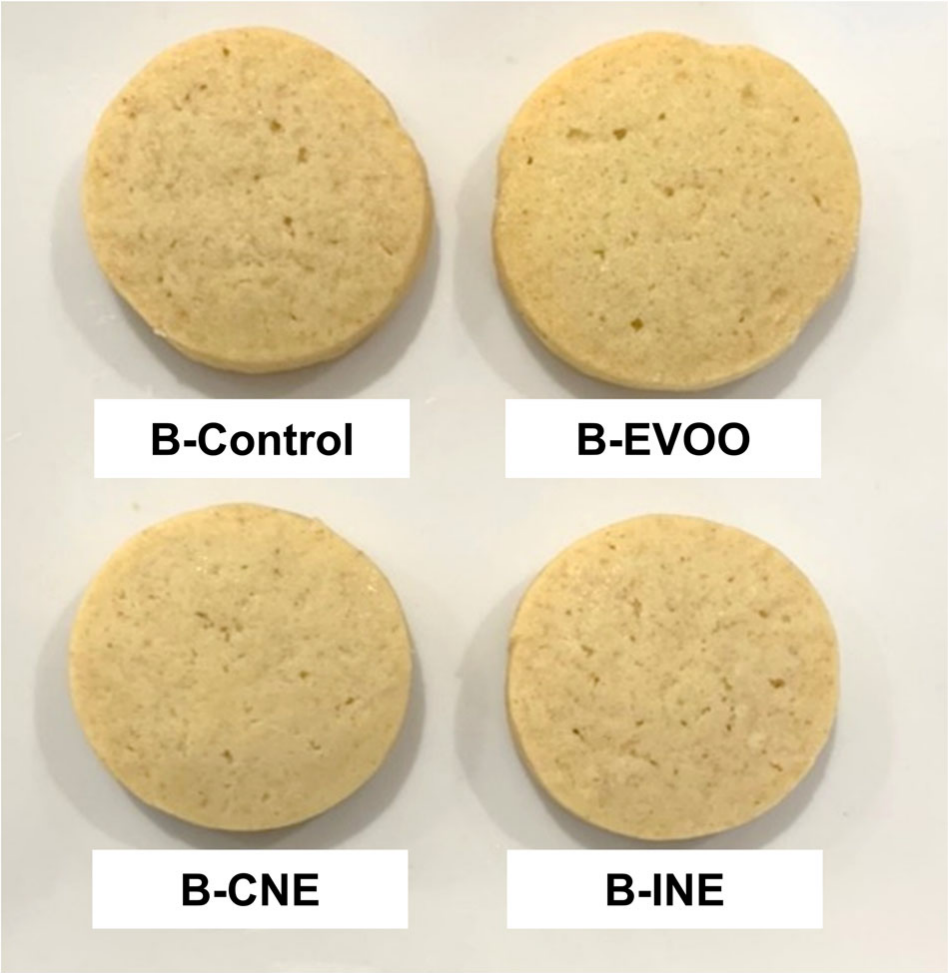
Visual appearance of biscuits (B-). Control is the biscuit made with butter; in EVOO 33% of the butter was replaced with Extra Virgin Olive Oil; CNE has 30% less saturated fat by replacing butter with a Complex Nano-Emulsion; INE has 30% less saturated fat by replacing butter with the individual ingredients of the complex nanoemulsion.

Biscuit quality can be summarised in two general aspects: the size and the bite^5^. Pareyt and Delcour^5^ and Manley^3^ explained that final biscuit dimensions depend on several factors: the dough spread onset, the dough spread rate during baking, and the set time. The dough spread onset is influenced by an increase in the mobility of gluten proteins as temperature increases and is influenced by the level of plasticiser (water) or anti-plasticiser (syrup). The dough spread rate during baking is defined by the levels of dissolved sugar and melted fat, or in other words it is defined by dough viscosity. The set time is defined by an ‘apparent’ glass transition of the protein network, increasing its viscosity and stopping cooking spreading. The height of the biscuit continues to increase due to gases formation until the structure collapses at the end of the baking process. The appearance of the four formulations of biscuits was as shown in Fig. 1.

Results of the SI of biscuits are presented in Table 2. B-Control, B-CNE and B-INE showed no statistical differences (*p* > 0.05) in terms of spreadability. On one hand, it could have been hypothesised that the fat reduction would have decreased the spread rate and the addition of water would have decreased the anti-plasticiser effect resulting in smaller biscuits. Previous work where the shortening was replaced by hydrocolloids (polydextrose, maltodextrin, inulin or whey proteins) in biscuits showed the diameter of the final products decreases due to elastic shrinkage tendency of these samples during baking^51,52^. On the other hand, the results from this study showed that the functionality of the CNE and its ingredients had a different effect on biscuit SI than other emulsions with HPMC. Previous studies in which oil in water emulsions with HPMC or ethyl cellulose were used to replace shortening in short dough biscuits reported a later set time for biscuits made with the emulsions^53^ together with higher biscuit diameter, thickness and SI values due to a softer and more fluid dough behaviour during baking^24^. However, the emulsions from the aforementioned studies, although using HMPC with similar degree of substitutions than the HPMC used in this study, contained higher amounts of oil (47–52%). Moreover, the rheological properties of the nanoemulsions stabilised by HPMC and lecithin could also explain the spreadability of the dough during baking. In a previous study the rheological behaviour during micro-baking was studied^39^; at low temperatures (20–40 °C) conventional emulsions and nanoemulsions stabilised by HPMC and lecithin showed a decrease in moduli values, which was related to a weakening effect in the intermolecular hydrogen bonds caused by the increased temperature. Then as temperature increased (40–65 °C), G’ and G” moduli increased, marking the starting point of the gelation temperature, during which the formation of strong hydrophobic interactions between HPMC molecules for the formation of a three-dimensional network took place^39^. This sol–gel transition could have control the spread of the dough during baking. The lower oil content (10%) and the combination of emulsifiers (HMPC and lecithin) used in this study to formulate the emulsions gave similar spreadability properties as butter, and as a consequence the strategy used in this study has given place to a similar spreadability behaviour in biscuits made with the nanoemulsion (B-CNE) than the ones with butter (B-control).

As expected, B-EVOO biscuits showed the highest (*p* < 0.05) SI. The higher content of liquid fat produced a softer dough with higher spreadability rate, higher final diameter and lower height. These results were in agreement with other studies, which observed that higher fraction of unsaturated fats lead to a higher spreadability index^21,54^, since the degree of saturation affects physical and thermal properties in fatty acids, specifically *cis* double-bonds in the acyl chain decrease the fat melting point. This characteristic of the oil decreases the viscosity of the dough, increasing the spreadability on set and rate.

#### Texture

During the baking process the viscoelastic dough changes into a solid with an aerated cellular structure and a characteristic texture^55^. The texture of biscuits is govern by the state of their principal components, fat continuity and size of inhomogeneities (gas cells); starch gelatinisation is limited due to the limited water content and low baking temperature^56^. Fracture strength and fracturability values of the biscuits are presented in Table 2. The results suggested that dough mechanical properties (Table 1) defined biscuits’ fracture strength. B-CNE and B-INE showed significantly higher (*p* < 0.05) fracture strength than B-Control; and B-INE showed the highest value among biscuit samples. When higher amount of fat is present in the system, it surrounds proteins and starch granules, isolating them and breaking the continuity of the structure^51,56^. In this study all dough formulations had the same water content but the presence of less fat in the system may have modify the accessibility to water and its distribution among hydrophilic components (sugar, starch, gluten and pentosans). When less fat is present in a dough formulation, less shortening effect is achieved, and more flour protein hydration takes place, giving rise to harder biscuits^3^. Starch hydration may also increase, but its gelatinisation will still be limited due to insufficient water content and low temperature and short baking time^56^. Biscuits in which 33% of butter was replaced by EVOO presented the lowest (*p* < 0.05) fracture strength values due to higher content of total fat and the lowest consistency of the oil at ambient temperature in comparison to butter or the emulsion. When comparing B-CNE showed similar (*p* > 0.05) fracturability to B-control, suggesting that these two samples had similar fracture-inducing defects (gas cells) and the CNE has the potential to be used as butter replacer for specific bakery applications.

### Microstructure

#### Confocal laser scanning microscopy (CLSM) of doughs and biscuits

**Fig. 2:**
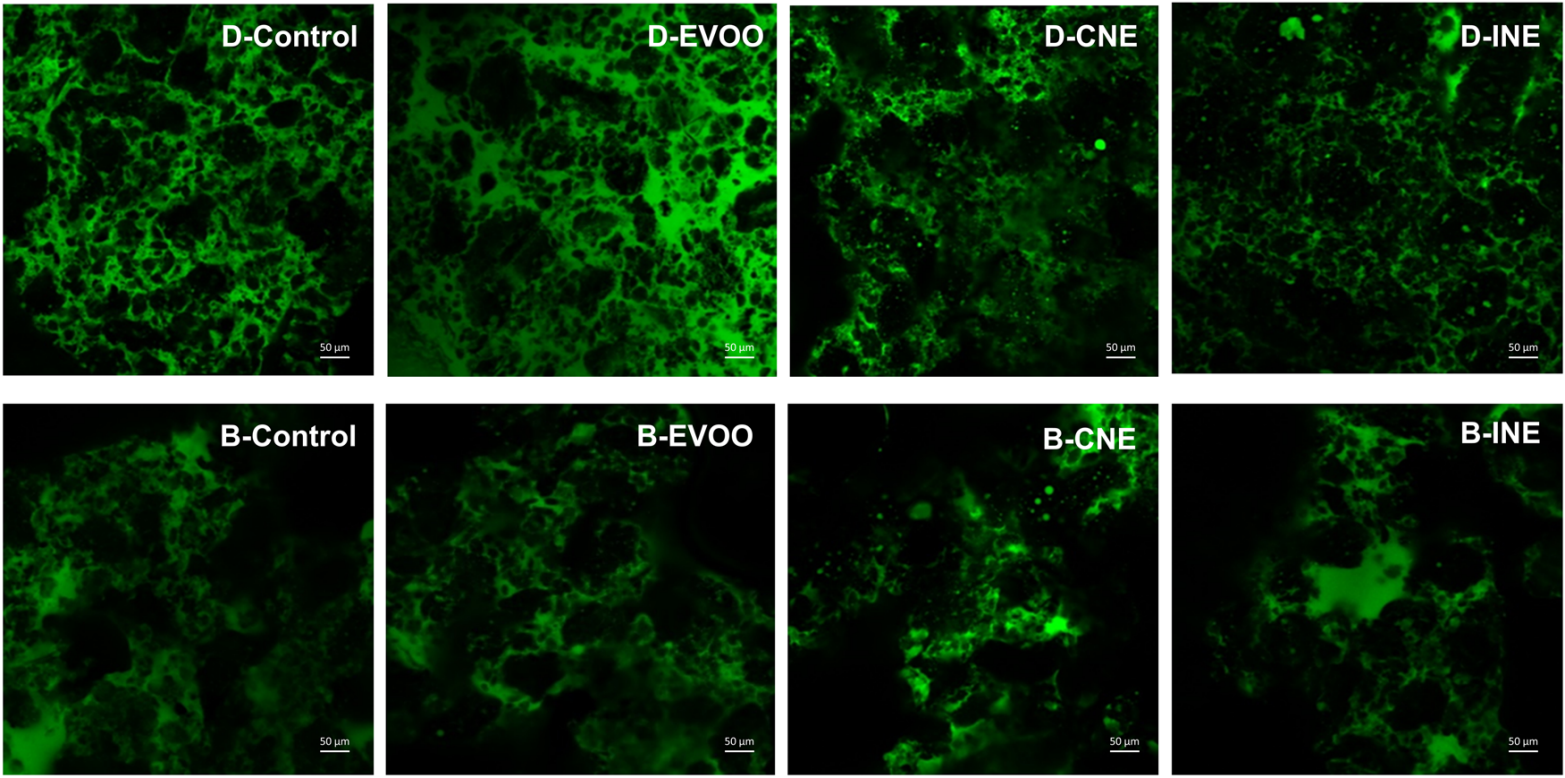
Confocal laser scanning microscopy (CLSM) of stained biscuit doughs (D-) and biscuits (B-) with Nile Red (fat in green and flour components in black). Control is the biscuit made with butter; in EVOO 33% of the butter was replaced with Extra Virgin Olive Oil; CNE has 30% less saturated fat by replacing butter with a Complex Nano-Emulsion; INE has 30% less saturated fat by replacing butter with the individual ingredients of the complex nanoemulsion. Scale bars: 50 µm.

The micrographs obtained by CLSM for doughs and biscuits are shown in Fig. 2. The samples were identified as fat globules in green and the starch granules in black. The dough of EVOO exhibited the highest fat globules dispersed in the dough among the samples, which this supported the result of the highest oil migration of D-EVOO as shown in Table 2. Regarding D-CNE and D-INE, there were fewer fat globules surrounding the starch granules than the D-Control and D-EVOO, which could be due to a lower fat content in the formulations. For the biscuit formulation, B-INE appears in the form of large fat globules dispersed in the matrix, whereas a more homogenous structure is apparent for B-CNE with more even distribution of fat and starch. This observation revealed that although there was a similar ingredient between CNE and INE, the individual ingredient added separately in B-INE may need more time to mix into the other ingredients. Overall, in the formulation of CNE, oil globules are observed dispersed more homogeneously in the dough and biscuit, what implies the complex nanoemulsion system has withstood the heat treatment.

#### Cryo-scanning electron microscopy (Cryo-SEM) micrographs of biscuits

**Fig. 3:**
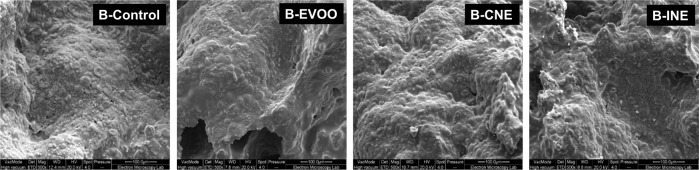
Cryo-SEM micrographs of short dough biscuits. B-Control is the biscuit made with butter; in B-EVOO, 33% of the butter was replaced with Extra Virgin Olive Oil; B-CNE has 30% less saturated fat by replacing butter with a Complex Nano-Emulsion; B-INE has 30% less saturated fat by replacing butter with the individual ingredients of the complex nanoemulsion.

Cryo-SEM micrographs of the short dough biscuits are shown in Fig. 3, revealing a microstructure comprised of starch granules embedded in a protein-sugar matrix, which was partially covered with fat^23,51^. B-Control and B-EVOO showed a higher smooth surface area, corresponding to more fat surrounding protein-starch aggregates. On the other hand, B-CNE and B-INE exhibited a more compact rough and uniform uneven structure. This result could be due to less fat surrounding starch granules, which has led to more exposed and visible starch granules in the surface of the biscuit structure. This could lead to B-CNE and B-INE having a different textural profile to the B-Control and B-EVOO, which had higher values of fracture strength as shown in Table 2.

#### Sensory profile of biscuits

**Fig. 4:**
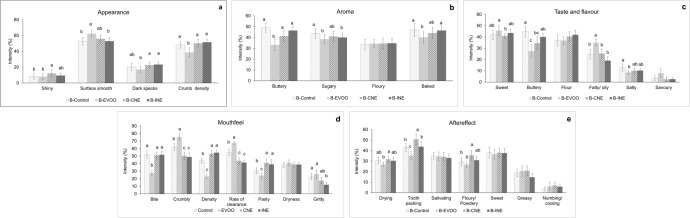
Mean values of sensory attributes for biscuits. **a** Appearance; **b** aroma; **c** taste and flavour; **d** mouthfeel and **e** aftereffect. Error bars represent ± standard error of the mean. Different letters indicate significant differences in the mean (*p* < 0.05) and no letter reflects no significant difference. B-Control is the biscuit made with butter; in B-EVOO, 33% of the butter was replaced with Extra Virgin Olive Oil; B-CNE has 30% less saturated fat by replacing butter with a Complex Nano-Emulsion; B-INE has 30% less saturated fat by replacing butter with the individual ingredients of the complex nanoemulsion.

The trained panel developed a consensus vocabulary of 28 attributes in 5 main modalities including appearance, aroma, flavour, mouthfeel and aftereffect as shown in (Table S.1). The results showed that 21 out of 28 attributes were found to differ significantly (*p* > 0.05) between samples. The sensory profiles of biscuits are presented in Fig. 4. As expected, due to their similar formulations, QDA evaluation identified that B-CNE and B-INE had more similar sensory attributes to B-Control than B-EVOO. There were no significant (*p* > 0.05) differences among the samples in terms of floury (aroma and taste), savoury (taste), dryness (mouthfeel), sweet (taste), greasy (aftereffect) and numbing/cooling (aftereffect).

In terms of the appearance (Fig. 4a), B-EVOO presented a significantly lower crumb density that the other three formulations and a significantly higher surface smoothness than B-Control. On the other hand, B-CNE and B-INE showed a similarly surface smooth, dark specks and crumb density as B-Control. The presence of HPMC in the formulation could be a factor to improve crumb density of B-CNE and B-INE because HPMC could develop a more stable system by preserving air bubbles during dough preparation and baking. This is in agreement with Bousquières et al.^57^, who stated that HPMC controlled the viscosity of cake batter and limited bubble loss during batter preparation. This paper also reported that during baking, methylcellulose (MC) and HPMC governed the sol-gel transition by forming hydrophobic bonds and undergoing concurrent starch gelatinisation, which affected on a wide range of cellular homogeneities in crumb. Moreover, B-CNE exhibited a significantly higher shiny appearance than B-Control and B-EVOO. This could be due to of the formation of a shiny HPMC layer coating the biscuit. HPMC is a widely used edible coating component to enhance the glossy appearance of fruit skins^58,59^.

When replacing butter in baked goods, it is important to consider several sensory properties of the final product: the aroma and flavour of butter; Maillard derived compounds and their sensory properties; the mouthfeel sensations from both the butter and structural changes in the baked good; the flavour release when changing the food matrix; and any new attributes introduced with the replacement ingredient. With regards to the aroma (Fig. 4b), a decrease in buttery, sugary and baked aroma was found in B-EVOO compared to B-Control, which is to be expected due to the reduction aroma compounds associated with butter and those derived from the Maillard reaction linked to baking and sugary aromas. However, there were no significant differences across aroma attributes between B-Control and B-CNE, indicating that aroma perception of B-CNE matched the same levels of B-Control. Although there was a reduction of butter in biscuits, there were no significant differences found for buttery, sugary, floury and baked aromas in B-CNE when compared to B-Control.

When assessing the taste and flavour (Fig. 4c), results suggested that a reduction of butter in biscuits by replacing with EVOO and CNE leads to a lower perception of buttery taste and flavour. While B-CNE and B-INE contained the same ingredients, the processing of those ingredients into a complex nanoemulsion did have an effect on some of the sensory properties of the biscuits. For example, where B-CNE did not vary from the control in sugary aroma, B-INE had a significantly lower intensity of sugary aroma (Fig. 4b). In addition, B-EVOO had significantly higher fatty/oily flavour than the other three formulations, which was expected due to higher amounts of oil in the composition leading to more aroma compounds associated with fatty/oily characteristics. B-Control was also found to be significantly more salty taste than B-EVOO. This result agrees with previous findings of Paneras et al.^60^, Romeih et al.^61^ and Chabanet et al.^62^, who observed greater saltiness intensity with a lower fat content in food products such as sausages, frankfurters and cheese, due to matrix effects.

Significant differences between samples were found across almost all mouthfeel attributes (Fig. 4d). Many of these differences found B-CNE and B-INE to be similar, with largest differences to B-EVOO. A significant decrease (*p* < 0.05) in firmness at first bite was found in the B-EVOO, which was in accordance with the instrumental texture results on fracture strength (Table 2). B-EVOO scored significantly higher for crumbly texture than all other samples; B-CNE and B-INE both scored significantly lower in crumbly texture than B-EVOO and B-Control. This indicates that B-CNE and B-INE were more compact and with a stronger continuous phase breaking into fewer crumbs during first bite. The presence of HPMC in the formula of B-CNE and B-INE could contribute to the similar bite to B-control and the lower crumbliness. This is supported by previous literature^23^, which reported that the microstructure of biscuits made with cellulose showed a dense matrix of stable fat globules, corresponding to the dispersed phase or oil globules, immersed in a continuous phase made up of water hydrated cellulose (a three-dimensional network of HPMC chains). Moreover, pastiness of biscuits significantly increased in B-CNE and B-INE, compared to B-Control. This increase in pastiness could be due to the tendency of HPMC to interact with water; when breaking the biscuits into smaller pieces during mastication, the continuous HPMC network is increasingly exposed to saliva, leading to a higher water absorption rate on the crumb pieces. This hypothesis could also explain the significantly higher tooth packing aftereffect (Fig. 4e) of B-CNE samples in comparison to other biscuits.

Overall, the sensory data showed that both B-CNE and B-INE were similar to B-control in many attributes. However, there were significant differences in shiny appearance, butter taste and flavour, and tooth packing between B-CNE and B-control, and significant differences in sugary aroma and gritty mouthfeel between B-INE and B-Control. Moreover, the finding of this study suggests that HPMC plays an important role on improving crumb density (appearance and mouthfeel) and bite of the biscuits. However, pasty and tooth packing of biscuits could be affected by HPMC, whereas EVOO could be a cause of fatty/oils in biscuits.

In conclusion, CNE doughs and biscuits were similar (*p* > 0.05) to the control formulation (butter) in terms of spreadability during baking, water activity and fracturability. CNE dough showed less oil migration during storage compared the other formulations. Although CNE biscuits showed a higher fracture strength, the trained panel did not find significant differences in crumb density and hardness on the first bite among CNE and the control biscuit. HPMC plays a major role on improving oil migration of dough and crumb density when an ingredient in biscuits. The use of the emulsion in the biscuit production was more effective and presented better results than the addition of the individual ingredients (B-INE). Quantitative descriptive analysis identified that B-CNE and B-INE had more similar sensory attributes to B-Control than B-EVOO. The trained panel did not find significant differences in crumb density and hardness on the first bite among CNE, INE and the control formulation. Overall, CNE which is the incorporation of a EVOO nanoemulsion stabilised with lecithin and HMPC has shown the potential to be used to reduce by 30% saturated fat and 25% total fat in short dough biscuits by replacing 33% of the butter with CNE.

## Methods

### Materials

Nanoemulsions were produced with extra-virgin olive oil (EVOO) (Napolina, Liverpool, England), soy lecithin (Louis Francois, Marne La Vallée, France) and HPMC powder (21.4% methoxy, 7.2% Hydroxypropyl, 26,000 g/mol; Carbosynth Ltd, 8&9 Old Station Business Park, UK). Regarding dough and biscuit preparation, raw materials included soft wheat flour (Heygates Ltd, Bugbrooke, England; composition data provided by the supplier: 9.1% protein; 80.9% carbohydrate; 13.5% moisture; falling number, 262 s), unsalted butter (82.0% fat, Co-op, Manchester, England), sugar (Silver Spoon White Granulated Sugar, Lynch Wood, Peterborough, English), milk powder (Nestlé, Vevey, Switzerland), salt (Morrisons brand, UK retail market), sodium bicarbonate (Hexeal brand, Norwich, UK), and ammonium bicarbonate (Atom Scientific Ltd, Manchester, England).

### Preparation of the nanoemulsion stabilised by lecithin and HMPC

Emulsions were prepared based on the methods described by Arancibia et al.^63^ and Taha et al.^64^ with some modifications. Firstly, a magnetic stirrer (ChemLab, Model SS3H) was used to prepare the aqueous phase dispersing soy lecithin (5% w/w) in water (83% w/w) at 200 rpm for 30 min at ambient temperature to ensure complete dispersion. Then, extra-virgin olive oil (EVOO) (10% w/w) was added to the aqueous phase during continuous stirring. The emulsions were homogenised with a high-speed homogeniser (Silverson, Model L4RT) at 10,000 rpm for 15 min.

To then prepare nanoemulsions according to the method described by Kampa et al.^39^, HPMC powder (2% w/w) was added to the conventional emulsion, and the mixture was stirred using a magnetic stirrer (ChemLab, Model SS3H) at 200 rpm for 3 h at ambient temperature and left 24 h at 4 °C to allow complete hydration of HPMC. The samples were then processed through a high-pressure homogeniser (HPH) (8.30H, Rannie, APV, Denmark) at 4×10^7 ^Pa for 1 cycle.

Nanoemulsion mean droplet diameter (MDD) and polydispersity index (PDI) were measured following the method described by Kampa et al.^39^ The particle size and polydispersity index of emulsions were determined by a dynamic light scattering (DLS) instrument (Zetasizer Nano ZS, Malvern Instruments Ltd., Worcestershire, UK). Emulsions had a pH of 4.24 ± 0.01, samples were diluted 100-fold with deionised water and agitated to avoid multiple light scattering effects. The dispersion was decanted into polystyrene cuvettes for measuring MDD and PDI. All the measurements were performed in triplicate. The droplet diameter (*z*-average diameter) of nanoemulsion stabilised by lecithin and HMPC was 192.08 ± 3.93 nm and the PDI 0.255 ± 0.013.

### Dough and biscuit preparation

Dough (D-) and biscuits (B-) were prepared according to Rodriguez-Garcia, et al.^51^ with specific adjustments. The ingredients used to produce the control biscuit doughs were (flour weight basis): soft wheat flour 100 g, butter 57 g, sugar 30 g, tap water 11.7 g, milk powder 0.58%, salt 0.2 g, sodium bicarbonate 0.2 g, ammonium bicarbonate 0.12 g.

In addition to the control formulation (100% butter), three more dough formulations were developed using the same amount of all ingredients except for the fat component. In a second formulation, 33% of the butter was replaced with EVOO, in a third formulation, 33% of the butter was replaced with the complex nanoemulsion (CNE); and in a fourth formulation 33% of the butter was replaced with the individual ingredients used for the formation of the nanoemulsion (INE). The final water content in all dough formulations was 22% (in flour basis) and the total fat content in the final biscuits was 27 % in B-Control, 27% in B-EVOO, 25% in B-CNE and 25% in B-INE. By replacing 33% of butter in the dough formulation, a 30% reduction in saturated fatty acids and a 25% reduction in total fat reduction was achieved in B-CNE and B-INE (Supplementary Table 1).

For the preparation of each dough formulation in triplicate, the butter and the saturated fat replacer were beaten in a mixer (Kenwood Chef XL 1200 W, UK) at speed 1 for 4 min. Then the sugar was added and mixed at speed 3 for 2 min until a homogeneous cream was formed. After this, milk powder was solubilised in the water and added to the mixer at speed 1 for 2 min. Finally, the flour, milk powder, sodium bicarbonate, ammonium bicarbonate and salt were added and mixed at speed 1 for 4 min. The resulting dough was allowed to rest in a heat-sealed polyethylene bag at 21 °C for 10 min. The dough was then sheeted to a thickness of 10 mm using a sheeting machine (Rondo STM-503 table-top reversible sheeter; Burgdorf, Switzerland). Once sheeted the dough was allowed to rest at 4 °C covered between 2 layers of parchment paper. After resting the dough was shaped in circular dough pieces of 30 mm of diameter. Dough pieces were packed in polypropylene bags and analysed within the following 24 h. Twenty dough pieces were placed onto a perforated tray and baked at 170 °C for 20 min with oven heating ratio at 90% top and 10% bottom (Polin 9 tray deck oven, Ing. Polin E C. S.p.A., Verona, Italy). After baking, biscuits were allowed to rest at 21 °C for 90 min. Biscuits were packed in polypropylene bags and analysed within the following 24 h.

### Dough characteristics

#### Texture: sphere penetration measurement

The texture of the dough pieces was analysed using a TA-XT2 texture analyser equipped with Texture Exponent software (Stable Micro systems Ltd., England). A sphere penetration test was performed following the method described by Rodriguez-Garcia et al.^51^ with some modifications. A stainless-steel spherical probe (P/0.75) was used to compress the centre of the dough piece at 60% strain deformation, with a trigger force of 0.049 N, and test speed 1 mm/s. The maximum force (N), denoted as the peak of the curve was measured. The test was performed in ten dough pieces of each dough batch.

#### Oil migration

Oil migration was determined according to Onacik-Gür et al.^22^ with some modifications. Five dough pieces were placed in a Petri dish with seven pre-weighed layers of tempura paper (Daiso oil-absorbing cooking paper, Japan) on the bottom and two layers on top. Samples were stored in temperature-controlled cabinets at 18 °C and at 30 °C for 2 h. After this time, the dough pieces were compressed in the TA-XT2 texture analyser with a cylinder probe (diameter 50 mm, P/50) up to 60% strain deformation for 10 s, with a trigger force of 0.98 N. The fat/oil migration was calculated as difference in the mass (g) of the paper before and after the test and expressed in percentage, considering the initial fat content of the sample. The analysis was conducted on three dough pieces per dough batch per formulation.

### Biscuit characteristics

#### Weight loss during baking, moisture and water activity

The weight loss (WL) of biscuits was recorded after resting at 21 °C for 90 min, which calculated by using the following Eq. (1):

1 $${{\mathrm{WL}}}\left( \% \right)=\left({W}_{{\mathrm{{dough}}}}-{W}_{{{\mathrm{biscuit}}}}/{W}_{{\mathrm{dough}}}\right)\times 100 \%$$

where *w* indicates weight (g). From each formulation, ten samples were weighted before (*W*_dough_) and after baking (*W*_biscuit_).

Biscuits were crumbled prior to moisture content and water activity (*a*_w_) measurements. Around 3 g of crumbs were placed on an aluminium tray and heated at 105 °C until constant weight in a Sartorius balance (M-Pact Series, Sarotorius Lab Instruments, Germany). For water activity measurements a Rotronic HygroLab meter (Rotronic, Zurich, Switzerland) was used. Measurements were performed in duplicate per batch of biscuits per formulation.

#### Spreadability Index

The spreadability index (SI) was calculated following the method described by Onacik-Gür and Żbikowska^19^. The height and diameter of 5 biscuits per batch were measured using an electronic caliper (RS PRO 150 mm Digital Caliper, RS PRO professional, Singapore). The spreadability index was calculated from the following Eq. (2):

2 $${\rm{SI}}={\rm{Diameter}}/{\rm{Height}}$$

#### Three-point bending test

The texture of the biscuits was analysed using a TA-XT2 texture analyser equipped with the Texture Exponent software (Stable Micro systems Ltd., England). Biscuits were broken using the three-point-bending rig probe (A/3PB) using the following conditions: supports 15 mm apart, the probe moved 5 mm with a test speed of 1 mm/s, and a trigger force of 0.05 N. The parameters measured were the force at break (N), and the distance at break (mm) as the fracture strength and the fracturability, respectively. The analysis was performed on ten biscuits per formulation batch.

### Microstructure

#### Confocal laser scanning laser microscopy (CLSM) of doughs and biscuits

Doughs and biscuits were observed using a Nikon A1-R confocal microscope (Nikon, Tokyo, Japan) following the method of Rodriguez-Garcia et al.^51^ with some modifications. An Ar laser line with wavelength of 488 nm was employed as light source to excite the fluorescent dye Nile Red (Fluka, Sigma- Aldrich, Missouri, USA) with *λ*_ex_ max 488 nm and *λ*_em_ max 515 nm. Nile Red was solubilized in PEG 200 at 0.1 g/L and was used as fluorescent dye to stain fat. Thin sections (<0.5 mm) of dough and biscuit samples were cut with a razor blade and placed on the central area of a microscope slide and a cover glass was place on top. Then, Nile Red solution was added to the surface of samples and let to diffuse into the sample for 10 min. The samples were observed using the ×20/0.8NA/Plan Apo VC objective lens and images were captured (512 × 512 pixel resolution) using the microscope software (NIS-Elements, 5.11.01, Nikon, Japan).

#### Cryo-scanning electron microscopy (Cryo-SEM) micrographs of biscuits

The microstructure of short dough biscuit was observed under Cry-SEM using a Quorum PP2000T cryo-SEM preparation chamber (Quorum Technologies Ltd, UK) coupled to a Quanta 600 FEG scanning electron microscope (FEI, UK) following the method used by Rodriguez-Garcia et al.^51^ with some modifications. The samples were frozen by immersion in liquid nitrogen slush (at −210 °C), where fracture of the sample took place and rapidly transferred under vacuum to the Quorum preparation chamber and the samples fractured at −190 °C before increase to −90 °C for 40 min to aid the sublimation of any surface ice. After that, the samples were coated with a thin layer of platinum for 80–120 s. Observations were carried out at 20 kV at temperature below −135 °C and magnification of ×500. Images were capture through the user interface (xT Microscope Server version 2.4) at 1024 × 943 pixel resolution.

#### Sensory analysis

Sensory profiling of biscuits was carried out using quantitative descriptive analysis (QDA). A trained sensory panel of ten people (*n* = 10; 8 female and 2 male) was used. All sensory evaluation was carried out in a temperature-controlled room (22 °C), in isolated booths, and under artificial daylight. The trained panel were provided with biscuits (10 g), which were prepared as described in Section’Dough and biscuit preparation’. The panel developed a consensus vocabulary of 28 attributes, which were grouped in 5 modalities: appearance, aroma, flavour, mouthfeel and after effect as outlined in Supplementary Table 2 in the results section. The purpose of the sensory profiling was to describe and quantify changes in biscuit descriptors occurring with change in formulation. All panellists scored in duplicate for each sample on 2 consecutive days. Samples were blind coded with three-digit numbers, and were presented in a monadic balanced order, with sample sets randomly allocated to panellists. Warm filtered water (~40 °C) and peeled raw carrot were provided for palate cleaning between samples. Unstructured linescales (0–100) with suitable anchors for each attribute were used (Compusense Cloud Software, Guelph, ON, Canada).

#### Statistical analysis

One-way analysis of variance (ANOVA) was performed using IBM SPSS 25 (Armonk, NY: IBM Corp, USA). Tukey’s HSD test was used to compare the mean values (*p* < 0.05) of weight loss during baking, water activity, textural properties, total count of crumb cells, cell size, spreadability index and colour parameters. A two-way analysis of variance was conducted on the influence of two independent factors: the formulation and storage time in the measurement of oil migration in biscuits. For sensory data, the QDA results were analysed using analysis of variance (ANOVA) in SenPAQ (version 5.01, Qi Statistics, Berkshire, UK). The panellists were fitted as random effects and the samples were fixed effects. The treatment effects (samples and assessors) were tested against the panellist by assessor interaction. Ad hoc tests for multiple means comparisons were carried out using Fisher’s least significant difference test (LSD) (*p* > 0.05).

### Reporting summary

Further information on research design is available in the Nature Portfolio Reporting Summary linked to this article.

## Supplementary information

Supplementary Tables

Reporting summary checklist

## Data Availability

The data presented in this paper are openly available in the University of Reading Research Data Archive at 10.17864/1947.000442 (accessed on January 2023).
