# Supplementary material for: Saturated fat replacement in short dough biscuits with HPMC and lecithin stabilised nanoemulsions
Source: NPJ Sci Food. 2023 Jun 7;7:26. doi: 10.1038/s41538-023-00202-5 (PMC10247766; doi:10.1038/s41538-023-00202-5)
Supplement: Supplementary file 1 — Supplementary Tables [file 41538_2023_202_MOESM1_ESM.pdf]

Supplementary Table 1. Nutritional composition of the biscuits (B-) (g/100g of biscuit). B-Control is the biscuit made with butter; in B-EVOO, 33% of the butter was replaced with Extra Virgin Olive Oil; in B-CNE has 33% of the butter was replaced with a Complex Nano-Emulsion; B-INE has 33% l of the butter was replaced with the individual ingredients of the complex nanoemulsion.

Biscuits	Fat	Saturated fat	Carbohydrates	Sugar	Protein	Fibre
B-Control	27	16.55	61	17	5	2
B-EVOO	27	12.70	61	17	5	2
B-CNE	20	11.63	61	17	5	3
B-INE	20	11.63	61	17	5	3

Values were calculated from the ingredient nutritional compositional information provided by suppliers.

Supplementary Table 2. Attributes, scale extremes and definitions used in the descriptive sensory analysis of the biscuits by a trained panel for the QDA.

Attribute	Scale extremes	Definitions
**Appearance**		
Shiny	low - high	Degree of shininess, which is the tendency of a surface to reflect light
Surface smoothness	none - extremely	Overall impression of the texture without roughness of the top surface of the biscuit.
Dark specks	none - lots	The number of dark specks within the biscuit.
Crumb density	low - high	The amount of air incorporated into the interior of the biscuit, observed visually upon breaking the biscuit in two.
**Aroma**		
Buttery	none - extremely	Intensity of a distinct butter aroma
Sugary	none - extremely	Intensity of sweet aroma, similar to table sugar.
Floury	none - extremely	Intensity of a distinct white wheat flour aroma
Baked	none - extremely	Intensity of bake aroma, similar to baked bakery
**Taste and Flavour**		
Sweet	none - extremely	Intensity of sweet taste (Sucrose)
Buttery	none - extremely	Intensity of a distinct butter flavour
Flour	none - extremely	Intensity of a distinct white wheat flour flavour
Fatty/ Oily	none - extremely	Intensity of a distinct vegetable oil flavour
Salty	none - extremely	Intensity of salty taste (sodium chloride)
Savoury	none - extremely	Intensity of umami taste (monosodium glutamate)
**Mouthfeel**		
Bite	soft – firm	Degree of force perceived at first bite

Crumbly	not – extremely	Degree of which the biscuit breaks into crumbs upon first bite
Density	low – high	Degree of how compact the inside of biscuit was upon chewing
Rate of clearance	slow- fast	Speed at which sample was able to be cleared from the mouth
Pasty	none - extremely	Degree to which the crumbs of biscuit begin to absorb saliva and form a paste
Dryness	none - extremely	Lack of lubrication in the mouth
Gritty	none - extremely	Degree of grainy or sandy texture
**Aftereffect**		
Drying	none - extremely	Degree to which 30 s after the biscuit was swallowed, your mouth still feels dry.
Tooth packing	none - extremely	Degree to which biscuit sticks to the surface of teeth
Salivating	none - extremely	Amount of saliva produced in the mouth
Floury/ powdery	none - extremely	Intensity of a powdery aftertaste
Sweet	none - extremely	Intensity of sweet aftertaste
Greasy	none - extremely	Degree to which the biscuit leaves an oily residue
Numbing/ cooling	none - extremely	Intensity of a numb sensation associated with a coolness in the mouth
